# Seasonal Variation in Fat Quality and Conjugated Linoleic Acid Content of Dairy Products from the Tropics: Evidence of Potential Impact on Human Health

**DOI:** 10.3390/foods6080061

**Published:** 2017-08-01

**Authors:** Juliana Côrtes Nunes, Monalisa Nilza Lole Ramalho da Silva, Daniel Perrone, Alexandre Guedes Torres

**Affiliations:** 1 Laboratory of Food Science and Nutritional Biochemistry, Institute of Chemistry, Federal University of Rio de Janeiro, Rio de Janeiro 21941-909, Brazil; jcortesnunes@gmail.com (J.C.N.); lolemonalisa@gmail.com (M.N.L.R.d.S.); danielperrone@iq.ufrj.br (D.P.); 2 Department of Food Science, School of Nutrition, Federal University of Rio de Janeiro State, Rio de Janeiro 22270-000, Brazil

**Keywords:** conjugated linoleic acid, atherogenicity index, cow’s milk fat, fatty acid composition, seasonality

## Abstract

Seasonal variation in conjugated linoleic acid (CLA) content and atherogenicity index (AI) of retail dairy products (whole milk, butter, and *prato*, a soft yellow cheese) from Brazil was investigated. CLA content of dairy products ranged from 0.55 to 1.53 g CLA/100 g fatty acids and was on average 25% higher during the rainy season compared to the dry season. Dairy products from the rainy season also had lower AI levels, indicating a lower risk of causing cardiovascular disease in consumers. This seasonality led to estimated seasonal variations of milk fat quality consumed by the population of southeastern Brazil, meaning 15% and 19% variation in daily intake of CLA and AI values, respectively. Dietary consumption of CLA (g/day) was greater in the rainy season, despite higher intake of dairy products during the dry season. We show that dairy products produced during the rainy season in Brazil are expected to be more beneficial to human health than are those produced during the dry season.

## 1. Introduction

Dairy products are rich food sources of conjugated linoleic acid (CLA) and saturated fatty acids that have an impact on human health. CLA is a group of linoleic acid isomers with conjugated double bonds [1] that exhibit biological activity such as anti-carcinogenicity, modulation of the immune system, and alterations in plasma lipid levels, insulin sensitivity, and body composition [2,3,4,5,6]. Cow’s milk fat is the major source of CLA in the human diet, with its content in milk ranging from 2 to 37 mg CLA/g lipid [7], and with *cis*-9, *trans*-11 as the major isomer (80% to 90% of total CLA) [8,9,10]. Conversely, saturated fatty acids, especially lauric (12:0), myristic (14:0), and palmitic (16:0) acids, are atherogenic, meaning that their intake promotes arterial wall hardening [11]. Accordingly, the atherogenicity index (AI) of dietary lipids [11] expresses the relation between these atherogenic saturated fatty acids on the one hand and unsaturated fatty acids, which are known to protect the cardiovascular system, on the other. Therefore, AI is expected to indicate the tendency of a dietary fat source to increase the risk of atherogenesis, and thus the risk of developing cardiovascular disease.

Fatty acid composition in dairy products can fluctuate widely according to several factors, especially seasonal environmental factors [12,13,14]. The CLA content of milk fat and its relation with seasonality have been reported for dairy products from several countries in the northern hemisphere [13,15,16,17,18]. Although seasonal variation in the fatty acid composition of dairy products from temperate climates has been extensively studied, this has not been the case for dairy products produced in regions with poorly defined annual seasons, such as those with a tropical climate. In these regions, the pattern of seasonality of dairy fat fatty acid composition might differ from that found in a temperate climate. Therefore, it is possible that the seasonality of fatty acid composition and CLA content in Brazilian dairy products differs from that of dairy products from European and North American countries with temperate climates. To the best of our knowledge, only two studies have examined CLA seasonal variation in Brazilian dairy products, and those studies were limited to investigating organic and pasteurized milk [19,20], whereas seasonal variation in the CLA content and fatty acid composition of a variety of commercial dairy products from Brazil has not been reported.

The aim of the present study was to determine seasonal variation of dairy fat composition and nutritional quality in commercial dairy products produced in a region with a sub-tropical climate (southeastern Brazil), which was used to estimate corresponding variation in daily CLA intake as well as in the AI of dairy fat consumed by the population. We hypothesized that seasonal variation in dairy fat composition in Brazil would not follow the same pattern observed in regions with a temperate climate. Seasonal variation in fatty acid composition might be accompanied by seasonality in dairy fat nutritional quality, which might be a risk factor for atherogenesis. This might be of special concern if seasonal variation in the CLA content and fat quality of dairy products leads to seasonality of dietary fat quality in general.

## 2. Materials and Methods

### 2.1. Samples

In the present study, three of the most consumed commercial brands from each of the most popular types of dairy products in Brazil were analyzed (Table 1), as follows: whole ultra-high temperature processed (UHT) milk, *prato* cheese (soft yellow cheese similar to the Danish cheese *danbo*), and butter from unfermented cream [10,21]. The selection of products’ commercial brands was based on their occurrence in hypermarkets chains in Brazil (*Carrefour*, *Extra,* and *Pão de Açúcar*), as previously described [22,23]. Availability of the selected brands in hypermarkets located in Rio de Janeiro capital (22°54′19.4112′′ S, 43°10′37.6608′′ W) was recorded between March and April 2010. The hypermarkets assessed by regular visits have stores located in all Brazilian southeastern states. The three commercial brands selected belong to the top ten dairy industries in Brazil and accounted together for 40% of total milk volume collected in Brazil in the year 2009 [24]. In addition, these brands are those that have the largest number of suppliers (29 farms, corresponding to 22% of registered national dairy suppliers). These suppliers are distributed in all geographic regions of Brazil [25].

After selection, the dairy products were acquired throughout one year in order to obtain representative samples from the four seasons (Table 1). All dairy products were produced approximately in the middle of each of the four seasons, and were acquired within one month from the fabrication date. Data on average rainfall during the period was considered in order to classify the season as rainy or dry. Rainfall was highest during spring and summer months (late September to late March in the southern hemisphere) and lowest during autumn and winter months (late March to late September) [26]. Thus, data for dairy products produced during the spring and summer were combined, and those two seasons were classified as the rainy season; likewise, autumn and winter were combined and classified as the dry season.

Samples from two production lots were acquired for each brand and type of dairy product. In order to obtain representative samples from each production lot, the contents of two packages of milk and three packages of butter or *prato* cheese were combined appropriately [10] All dairy products samples were in their individual original industrial packages, and were not fractionated locally at the market. Milk samples were purchased in 1 liter carton packages, butter samples in 0.2 kg tablets and *prato* cheese in 0.5 kg vacuum-sealed plastic wraps. A total of twelve samples of dairy products for each season, with a total of forty-eight samples in one year, were used. The samples were kept at −20 °C under N_2_ until analysis.

### 2.2. Fatty Acids Analysis

#### 2.2.1. Lipid Extraction and Derivatization of Fatty Acids

Total lipids were extracted with hexane:isopropanol 3:2 (*v*/*v*) [27] with appropriate adaptations for each food matrix [10]. Fatty acids were methylated through base-catalyzed transesterification [28] in order to avoid CLA isomerization. Briefly, lipid extracts were dissolved in *n*-hexane followed by addition of a NaOCH_3_ solution in methanol (0.3 mol/L) and heating at 50 °C in a water bath for 10 min. Then, 10% HCl (*w*/*v*) in methanol was added and heated at 80 °C in a water bath for 10 min. The resulting fatty acid methyl esters (FAME) were extracted with hexane by centrifugation after addition of 28% NaCl solution (*w*/*v* in water). The upper hexane layer was evaporated, and the FAME were suspended in hexane and stored at −20 °C until analysis by gas-chromatography (GC). All samples were analyzed in triplicate.

#### 2.2.2. Fatty Acid Composition of Dairy Products

FAME were analyzed in a GC-2010 gas chromatograph (Shimadzu, Tokyo, Japan) equipped with a flame ionization detector (FID), a split/splitless injector (with a 1:30 split ratio), and a polyethylene glycol capillary column (30 m, 0.32 mm i.d., 0.25 μm film thickness; Omegawax-320, Supelco Co., Bellefonte, PA, USA). In order to confirm CLA isomer distribution in dairy fat, two samples of butter were analyzed in a highly polar (88%-cyanopropyl) polysiloxane capillary column (60 m, 0.25 mm i.d., 0.25 μm film thickness; Agilent, Santa Clara, CA, USA). Detailed gas-chromatographic operational conditions were previously reported [29]. Relative retention times of commercial standards (37 FAME mix and CLA methyl esters; Sigma-Aldrich, São Paulo, Brazil) were used for identification of gas-chromatographic FAME peaks. The method described by Torres et al. (2002) [30] and the equivalent chain lengths system [31] were used as complementary tools for the identification of FAME peaks. The areas of the peaks were corrected by the theoretical response factors of Ackman and Sipos for the FAME responses of the flame ionization detector as described by Wolff et al. (1995) [32]. Content for an individual fatty acid was expressed as g/100 g total fatty acids. The AI of the dairy products was calculated based on the contents of lauric (C12:0), myristic (C14:0), and palmitic (C16:0) acids and the sum of unsaturated fatty acids (Equation (1)) [11]. All the materials used for analysis of fatty acids were rinsed with alcoholic KOH (2.5%, *w*/*v*) and soaked in HNO_3_ solution (13%, *w*/*v*) for 2 h. All solvents were of chromatographic grade from Tedia (Fairfield, OH, USA). HPLC grade water (Milli-Q System, Millipore, Bedford, MA, USA) was used throughout the experiments.

(1) AI=(C12:0+(4×C14:0)+C16:0)/ΣUFA

where, *AI*: atherogenicity index; C12:0, C14:0, and C16:0 are contents (g/100 g fatty acids) of these saturated fatty acids; Σ*UFA*: sum of contents of all unsaturated fatty acids.

### 2.3. Estimated Intake of CLA and Dairy Fat Quality

According to data from the Brazilian National Household Food Budget survey [21] and calculated annual per capita consumption, UHT milk, *prato* cheese, and butter represent 86% of the total dairy products and 71% of the total dairy fat consumed by the Brazilian population [10,21]. Thus, estimated consumption of CLA from intake of dairy products during each season was estimated by multiplying the CLA content of each dairy product by the estimated consumption of that food type during each season by the population of southeastern of Brazil [33]. This estimate was made possible for that Rossato et al. [33] assessed the intake of food groups throughout one year (with seasonal assessments) in southern Brazil. Seasonal variation in dairy fat quality consumed in southeastern Brazil was assessed using the AI of consumed dairy fats and estimated as described for CLA.

Concerning representativeness of the present assessment, southeastern Brazil is a region of over 70 million people, representing roughly 40% of the country’s population, and comprises the states of Espírito Santo, Minas Gerais, Rio de Janeiro, and São Paulo.

### 2.4. Statistical Analysis

Descriptive analysis was used to calculate means, standard deviations, and standardized coefficients of kurtosis and skewness. One-way analysis of variance (ANOVA) with Tukey’s *post hoc* test was used to investigate the influence of seasonality on fatty acid composition and *cis*, *trans* CLA content. An unpaired *t*-test was used to compare the fatty acid composition and the *cis*, *trans* CLA content of dairy products between the dry and rainy seasons. Pearson correlation analysis was performed to investigate associations between CLA and selected fatty acids. Data were analyzed with GraphPad Prism 5.0 (GraphPad Software, San Diego, CA, USA). Two-sided *p* values <0.05 were considered as statistically significant.

## 3. Results

### 3.1. Fatty Acid Composition and Atherogenicity Index of Brazilian Dairy Products

Thirty-six fatty acids were identified in the Brazilian dairy products. Contents of major fatty acids (content >0.25% of total fat) of each dairy product type produced during both the rainy and dry seasons are presented in Table 2. We observed significant seasonal variation in the fatty acid composition of Brazilian dairy products, which agrees with previous reports from regions with a temperate climate [13,17].

When data from all dairy products were combined, the levels of total saturated fatty acids (SFA), which included fatty acids synthesized de novo such as 14:0 and 16:0, were lower in the rainy season than in the dry season. Conversely, the sum of polyunsaturated fatty acids (PUFA) was higher in the rainy season than in the dry season (Table 2).

**Figure 1:**
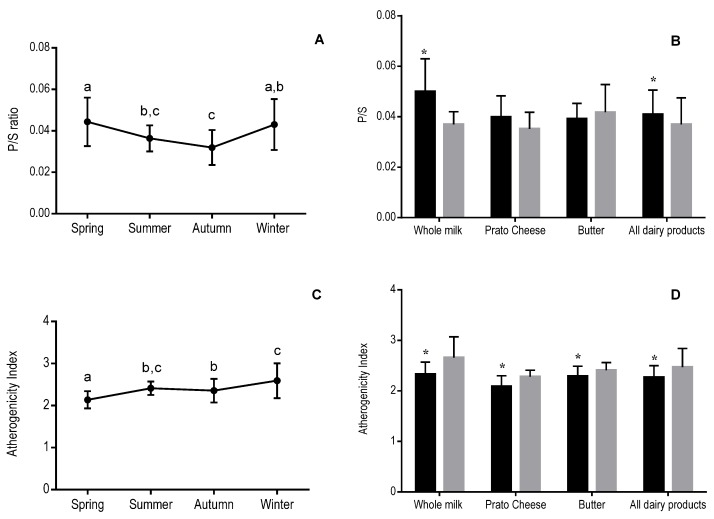
Seasonal variation in polyunsaturated-to-saturated fatty acid ratio (P/S; **A**), atherogenicity index (AI; **C**), and effects of rainy and dry seasons on P/S (**B**) and AI (**D**) of Brazilian dairy products. Data from all dairy products were combined. AI = (C12:0 + (4 × C14:0) + C16:0)/(sum of unsaturated fatty acids). (**A**,**C**): Data points and error bars represent means and standard deviations, respectively. Means sharing a lowercase letter are not significantly different by ANOVA with Tukey’s post-test (*p* < 0.05). (**B**,**D**): Spring and summer were the rainy seasons (■) and autumn and winter were the dry seasons (■). * Significantly different from the dry season value by *t* test (*p* < 0.05).

The polyunsaturated to saturated fatty acid ratio (P/S) of the dairy products varied over the four seasons (Figure 1A) and, when data for all products were combined, between the rainy and dry seasons (Figure 1B). The AI of each dairy product in each season was calculated based on the contents of lauric (C12:0), myristic (C14:0), and palmitic (C16:0) acids and the sum of unsaturated fatty acids [11]. When data from all dairy products were combined, the AI of Brazilian dairy products varied over the four seasons (Figure 1C). However, the pattern of seasonal variation in AI was more consistent when the seasons were grouped into rainy and dry seasons, with lower values of AI observed during the rainy season than during the dry season for each product type (Figure 1D).

AI was more sensitive than P/S ratio to the effects of seasonality on the fatty acid composition of dairy products, since the former differed significantly between the rainy and dry seasons for each of the individual dairy products, while the latter differed only when all products were evaluated together (Figure 1B,D).

### 3.2. Seasonal Variation of CLA Content of Brazilian Dairy Products

We report for the first time the variation of the *cis*, *trans* CLA content of three widely consumed Brazilian dairy products over four seasons in a 12-month period. The average CLA content of these dairy products ranged from 0.55 to 1.53 g CLA/100 g of fatty acids (FA) during the year (Table 2), which is roughly similar to the average CLA content previously reported for European dairy products, which ranged from 0.40 to 1.70 g CLA/100 g FA [18,34,35]. Although the analytical method employed in the present study did not separate *cis, trans*/*trans*, *cis* isomers, which include *cis*-9, *trans*-11, *trans*-7, *cis*-9, and *trans*-8, *cis*-10 CLA isomers, the analysis of two samples of Brazilian butter from spring in a highly polar cyanopropyl polysiloxane capillary column showed that 90% of total CLA consisted of the *cis*-9, *trans*-11 isomer. This result is consistent with those of previous reports for dairy products from Brazil [10] and other countries [9,36].

**Figure 2:**
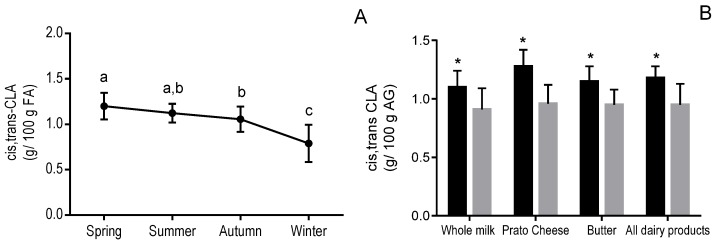
Seasonal variation (**A**) and effects of rainy and dry seasons (**B**) on the content of CLA in Brazilian dairy products. Data from all dairy products were combined. (**A**): Data points and error bars represent means and standard deviations, respectively. Means sharing a lowercase letter are not significantly different by ANOVA with Tukey’s post-test (*p* < 0.05). (**B**): Spring and summer were the rainy seasons (■) and autumn and winter were the dry seasons (■). * Significantly different from the dry season by *t* test (*p* < 0.05).

The CLA content of Brazilian dairy products varied over the four seasons (Figure 2). CLA content was lower during winter and autumn (Figure 2A), which agrees with expected changes in the cows’ diets between autumn-winter and spring-summer. This seasonal variation was evident when data from all dairy products were taken together (Figure 2A), with a 29% higher average CLA content in summer than in winter. Our finding that the CLA content of dairy products was lowest during the winter agrees with previous studies that found that the average CLA concentration in milk fat was approximately 40% higher in summer than in winter [13,18,20]. In a previous report on milk produced in Brazil [19], the CLA content of organic milk was 57% higher in summer than in winter. However, CLA did not vary seasonally in conventional milk [19].

Although the general pattern of seasonal variation in CLA content occurred as expected, the CLA content of Brazilian butter was not lower in the winter than in the summer or spring (data not shown). However, the seasonal variation of CLA in dairy products was more consistent when the individual seasons were grouped into rainy or dry seasons (Figure 2B). As expected, the CLA content of Brazilian dairy products was lower in the dry season compared to the rainy season, consistent with expected seasonal changes in the cows’ diet.

### 3.3. Seasonal Variation in Estimated CLA Intake and Atherogenicity Index of Dairy Products Consumed in Southeastern Brazil

**Figure 3:**
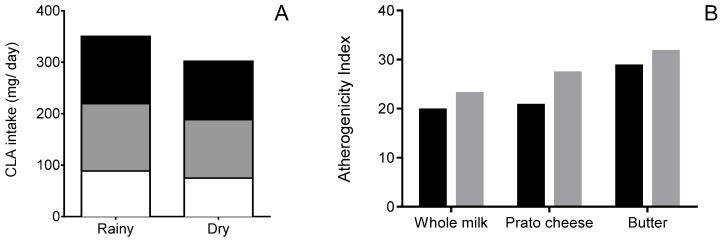
Estimated per capita intake of CLA (**A**) and atherogenicity index (**B**) associated with consumption of milk fat from dairy products in southeastern Brazil. Spring and summer were the rainy seasons and autumn and winter were the dry seasons. (**A**): Dairy products: ■ Butter; ■ *prato* cheese; ☐ whole milk. (**B**): ■ Rainy season; ■ Dry season.

Dietary intake of CLA depends on the habitual diet of the population and on the CLA content of dairy products, which are its major source. Rossato et al. (2015) [33] observed seasonal variation in food consumption among the population of southeastern Brazil. In their study, intake of whole milk, whole cheese, and animal fat was higher during the autumn when compared to other seasons. Using their data on food intake combined with our data on variation in CLA content of dairy products, we estimated that daily intake of CLA is 15% higher during the rainy season (347 mg/day) compared to the dry season (303 mg/day) (Figure 3A). Butter is the major dairy food source of CLA in both seasons, contributing approximately 40% of total CLA intake, followed by *prato* cheese (35%), and whole milk (25%) (Figure 3A). The CLA content of dairy products was the major factor contributing to higher intake of CLA in the rainy season, despite the higher intake of dairy products during autumn, which is a dry season. The estimated AI from milk fat consumed in southeastern Brazil was on average 19% higher in the dry season compared to the rainy season (Figure 3B). Therefore, seasonal variations of CLA content and AI of dairy products are likely to affect the intake of CLA and the AI of dairy products consumed by the population of southeastern Brazil.

## 4. Discussions

In the present study, we show that the CLA content and atherogenicity index (AI) of dairy products from a tropical region vary between the rainy and dry seasons, with higher CLA levels and lower AI values found in dairy products produced during the rainy season. This seasonality leads to variation in the estimated daily intake of CLA from consumption of dairy fat, as well as the AI of that fat. In southeastern Brazil, per capita dietary consumption of CLA (g/day) and AI were greater during the rainy season, despite the higher intake of dairy products during the dry season. These results suggest that dairy products produced during the rainy season (spring-summer) in Brazil should be more beneficial to human health than those produced in the dry season (autumn-winter).

The seasonality observed in fatty acid composition in this study agrees with results from previous studies done in regions of temperate climate, which showed higher PUFA and CLA and lower SFA in products produced in the summer than those produced in the winter.

In most of Brazil, especially in regions that produce milk and other dairy products such as the southeast, summer and spring are typically rainy while winter and autumn are drier seasons, when rainfall averages are typically low [37]. Consequently, it is expected that fresh pasture and forage, which are rich in PUFA, were more available during the rainy season. Feeding dairy cows with diets containing high levels of PUFA inhibits de novo fatty acid synthesis in mammary tissue [38]. Additionally, PUFAs are transformed into CLA by rumen biohydrogenation, so feeding high levels of PUFA leads to an increase in the CLA content of milk fat. Because de novo fatty acid synthesis in mammary tissue is also inhibited by CLA [39], milk PUFA and CLA are often negatively associated with milk SFA. In the present study, CLA and fatty acids synthesized de novo were negatively correlated (*r* = −0.39, *p* < 0.000) when all dairy products were taken together. The pattern of seasonal variation in the CLA content of the investigated dairy products was similar to that of previous reports [13,17,18,40]. Altogether, our findings are consistent with the hypothesis that the CLA content of Brazilian dairy products varies according to rainfall rather than with average temperature.

The polyunsaturated to saturated fatty acid ratio (P/S) is a dietary index associated with the development of cardiovascular diseases related to serum cholesterol levels [41,42], especially atherosclerosis. Raising dietary P/S has been recommended by the American Heart Association for the prevention of cardiovascular disease [42]. Therefore, it is possible that dairy fat quality, in relation to the prevention of atherogenesis and cardiovascular disease, might vary seasonally. In this study, P/S ratio did not vary consistently between each of the four seasons, because average values of summer and autumn, and spring and winter were more similar between each other. This might be seen as a limitation of our study and was possibly caused by a buffering effect of random variation between brands and milk suppliers, because these and other factors influencing on milk fatty acid composition were not controlled for. However, if there is seasonality on the effects of fatty acid composition of commercial dairy products on consumers’ health, any causal relationship might be attenuated by these and other random factors. It seems that in the present study the P/S ratio was not a sensible marker of dairy fat quality seasonality, when the four seasons were considered independently, although it was consistent and sensible when grouped as rainy and dry seasons.

The AI is also of interest to human nutrition, and is related to the risk of development of cardiovascular diseases [43]. The consumption of dairy products with lower AI values leads to lower levels of total cholesterol and LDL-cholesterol in human plasma [43]. In the present study, both P/S and AI results indicated that Brazilian dairy products produced in the rainy season had a more favorable fatty acid composition with regard to human health. However, because AI was more sensitive than P/S to the effects of seasonality on dairy fat composition, the former might be a better index for the assessment of dairy fat nutritional quality and might also be used as an additional tool to guide milk fat consumption.

The seasonality of CLA and AI in Brazilian dairy products led to variation in estimated daily intake of CLA and AI from dairy fat. If seasonality of dairy fat quality truly impacts consumers’ health, then our findings may explain the fact that, both in Brazil [44] and in regions with a temperate climate such as the US [45], the UK, and Japan [46], the prevalence of dyslipidemia varies seasonally, with plasma total cholesterol and LDL-cholesterol higher during autumn and/or winter and lower during spring and/or summer.

Assessing the global quality of dairy fat as a proxy of the risk of cardiovascular diseases is a complex issue, and still a matter of debate [47]. In a recent review, dairy fat intake was considered neutral to cardiometabolic disease risk in humans [47]. However, in a large prospective study in Brazil (ELSA-Brazil), dairy fat intake was protective to cardiometabolic disease risk [48], and was negatively associated with newly diagnosed cases of diabetes [49], even when dairy fat was served in desserts and fast foods. These data highlight to the importance of habitual diets in a particular group, in order to correctly address the effects of a given food group to a health-related target. In this sense, it might be speculated that the protective effect of dairy fat intake observed in these population studies in Brazil are in some way related to the low habitual intakes of dairy products, but this hypothesis remains to be confirmed. The use of dietary indices that consider several fatty acids with known impacts on health is helpful in addressing these controversies, because these indices tend to balance the effects of individual fatty acids on health. It should not be discarded the potential protective effects of other components in dairy products, such as short-chain fatty acids, vitamin D, peptides, pro-biotic microorganisms, which may also influence the overall effects of dairy fat intake on human health.

It should be noted that our data are based on estimates of intake, and that actual dairy fat intake was not assessed directly. Nevertheless, based on our data and on data from other studies showing the seasonality of dairy product CLA content and of dyslipidemia [15,16,44,46], we hypothesize that an association exists between the prevalence of dyslipidemia and seasonal variations in the CLA content and AI of dairy products. It is also possible that other factors that vary seasonally, such as habitual diets, physical activity, hemodilution, and vitamin D biosynthesis also contribute to seasonal changes in blood lipids, as discussed elsewhere [44]. In any case, the hypothesis that seasonal variations in the CLA content and AI of dairy products may have consequences to human health merits further investigation.

## 5. Conclusions

Our data suggest that Brazilian dairy products produced during the rainy season are more beneficial to consumers’ health than those produced during the dry season, as the former have higher CLA levels and lower values of the atherogenicity index. Our findings should be taken into consideration in efforts to improve dietary guidelines and diet planning for individuals with increased risk of cardiovascular disease. It is possible that adding PUFA-rich vegetable oils to cow diets during the dry season might improve the nutritional quality of dairy products produced in that season and contribute to the prevention of cardiovascular disease in consumers of dairy products.

## Figures and Tables

**Table 1:** Details of samples of commercial dairy products (whole milk, butter from unfermented cream, and *prato* cheese) acquired in hypermarkets.

Dairy Product	Brand	Lot Number and Production Year or Fabrication Date	Dairy Product	Brand	Lot Number and Production Year or Fabrication Date
*Winter*			*Summer*		
UHT milk *	A	06IA02-2007	UHT milk	A	5IP12-2008
UHT milk	A	02SC03-2007	UHT milk	A	02LR13-2008
UHT milk	B	03ACL-2007	UHT milk	B	C2D1721-2008
UHT milk	B	LCZL01-2007	UHT milk	B	CZE4221-2008
Butter	A	21 August 2007	Butter	A	27 December 2007
Butter	A	3 September 2007	Butter	A	29 January 2008
Butter	C	1 August 2007	Butter	C	17 February 2008
Butter	C	7 August 2007	Butter	C	9 February 2008
*Prato* cheese	D	1 October 2007	*Prato* cheese	D	10 January 2008
*Prato* cheese	D	1 October 2007	*Prato* cheese	D	10 January 2008
*Prato* cheese	E	2 October 2007	*Prato* cheese	E	15 March 2008
*Prato* cheese	E	2 October 2007	*Prato* cheese	E	15 March 2008
*Spring*			*Autumn*		
UHT milk	A	06VV15-2007	UHT milk	A	WC0256-2008
UHT milk	A	3031922-2007	UHT milk	A	EA0248-2008
UHT milk	B	CZJ06-2007	UHT milk	B	L09B02-2008
UHT milk	B	053-2007	UHT milk	B	L090105-2008
Butter	A	6 November 2007	Butter	A	10 May 2008
Butter	A	26 November 2007	Butter	A	19 May 2008
Butter	C	21 November 2007	Butter	C	2 May 2008
Butter	C	22 November 2007	Butter	C	8 May 2008
*Prato* cheese	D	28 November 2007	*Prato* cheese	D	12 May 2008
*Prato* cheese	D	28 November 2007	*Prato* cheese	D	12 May 2008
*Prato* cheese	E	5 November 2007	*Prato* cheese	E	23 May 2008
*Prato* cheese	E	5 November 2007	*Prato* cheese	E	23 May 2008

* UHT: ultra-high temperature pasteurization processed milk.

**Table 2:** Content of the major fatty acids and fatty acid classes in dairy products produced in southeastern Brazil during the rainy and dry seasons. Values are g/100 g total fatty acids.

Fatty Acids and Fatty Acid Classes	Whole UHT Milk		*Prato* Cheese		Butter		All Dairy Products	
	Rainy	Dry	Rainy	Dry	Rainy	Dry	Rainy	Dry
Total CLA	1.10 * ± 0.14	0.91 ± 0.28	1.28 * ± 0.14	0.96 ± 0.16	1.15 * ± 0.07	0.95 ± 0.21	1.16 * ± 0.13	0.93 ± 0.23
Saturated								
14:0	11.2 * ± 0.69	12.7 ± 1.39	10.8 ± 0.55	11.2 ± 0.49	11.2 * ± 0.57	11.5 ± 0.25	11.0 * ± 0.60	11.9 ± 1.18
16:0	30.5 ± 1.47	30.7 ± 0.86	29.1 * ± 1.39	30.7 ± 0.77	30.4 * ± 1.18	31.4 ± 1.94	30.0 * ± 1.39	30.9 ± 1.49
18:0	13.0 * ± 0.92	10.9 ± 0.50	13.1 * ± 0.78	12.1 ± 0.44	12.9 * ± 0.73	11.7 ± 0.56	13.0 * ± 0.80	11.43 ± 0.69
Total SFA	62.6 ± 2.04	63.8 ± 2.66	60.9 ± 1.71	62.2 ± 1.04	62.0 ± 3.19	63.2 ± 1.37	62.0 *± 2.57	63.3 ± 2.03
Total BCFA	3.01 * ± 0.43	3.36 ± 0.20	3.23 * ± 0.25	2.79 ± 0.56	3.07 * ± 0.29	2.87 ± 0.21	3.05 ± 0.36	3.05 ± 0.40
Monounsaturated								
14:1	0.96 * ± 0.07	1.26 ± 0.07	1.07 * ± 0.08	1.18 ± 0.11	1.04 * ± 0.13	1.11 ± 0.11	1.02 * ± 0.11	1.19 ± 0.12
16:1	1.79 * ± 0.17	2.06 ± 0.16	1.87 ± 0.13	1.97 ± 0.19	1.86 * ± 0.17	2.02 ± 0.14	1.84 *± 0.16	2.03 ± 0.16
18:1	26.1 * ± 1.04	24.7 ± 2.41	27.9 * ± 1.61	26.6 ± 1.09	26.8 * ± 1.46	25.9 ± 0.86	26.8 * ± 1.49	25.5 ± 1.84
Total MUFA	29.8 ± 1.25	29.0 ± 2.65	31.9 ± 1.63	30.9 ± 1.17	30.5 ± 1.55	30.0 ± 0.87	30.5 * ± 1.63	29.7 ± 1.98
14:1/14:0 ratio (%)	8.66 * ± 0.51	10.2 ± 1.33	9.99 * ± 0.42	10.4 ± 0.26	9.22 ± 1.11	9.63 ± 0.99	9.16 * ± 0.93	9.99 ± 1.10
Polyunsaturated								
18:2*n*-6	2.06 * ± 0.48	1.54 ± 0.34	1.64 ± 0.29	1.62 ± 0.37	1.66 * ± 0.19	2.07 ± 0.46	1.80 ± 0.39	1.76 ± 0.47
18:3*n*-3	0.55 * ± 0.11	0.42 ± 0.06	0.49 * ± 0.14	0.34 ± 0.05	0.47 ± 0.07	0.46 ± 0.19	0.50 * ± 0.11	0.42 ± 0.13
Total PUFA	2.72 * ± 0.80	2.16 ± 0.38	2.46 * ± 0.45	2.10 ± 0.31	2.44 ± 0.24	2.63 ± 0.71	2.55 * ± 0.58	2.33 ± 0.57

Results are presented as mean ± standard deviation. Total saturated fatty acids (SFA), sum of 8:0, 10:0, 12:0, 14:0, 15:0, 16:0, 18:0, 20:0, and 22:0. Total branched chain fatty acids (BCFA), sum of *iso*11:0, *iso*13:0, *iso*14:0, *iso*15:0, *anteiso*15:0, *iso*17:0, *anteiso*17:0, *iso*18:0. And 18:1, consisting of the sum of all 18:1 isomers. Total monounsaturated fatty acids (MUFA), sum of 12:1, 14:1, 15:1, 16:1, 17:1, 18:1, and 20:1*n*-9. 14:1/14:0 ratio (%): content ratio of 14:1/14:0 × 100%. Total polyunsaturated fatty acids (PUFA), sum of 18:2*n*-6, 18:3*n*-6, 18:3*n*-3, and 20:3*n*-3. * Significantly different from the dry season value by *t* test (*p* < 0.05).

## Abbreviations

AI	atherogenicity Index
BCFA	branched chain fatty acids
CLA	conjugated linoleic acid
FA	fatty acid
MUFA	monounsaturated fatty acid
PUFA	polyunsaturated fatty acid
SFA	saturated fatty acid
